# Identifying Telemedicine Services to Improve Access to Specialty Care for the Underserved in the San Francisco Safety Net

**DOI:** 10.1155/2011/523161

**Published:** 2011-12-10

**Authors:** Ken Russell Coelho

**Affiliations:** ^1^Global Health Sciences, University of California, San Francisco, 50 Beale Street, Suite 1300, San Francisco, CA 94143, USA; ^2^Performance Improvement and Patient Safety Division, San Francisco General Hospital & Trauma Center, 1001 Potrero Avenue, Bldg 20, 3rd Floor, Suite 2300, San Francisco, CA 94110, USA

## Abstract

Safety-net settings across the country have grappled with providing adequate access to specialty care services. San Francisco General Hospital and Trauma Center, serving as the city's primary safety-net hospital, has also had to struggle with the same issue. With *Healthy San Francisco*, the City and County of San Francisco's Universal Healthcare mandate, the increased demand for specialty care services has placed a further strain on the system. With the recent passage of California Proposition 1D, infrastructural funds are now set aside to assist in connecting major hospitals with primary care clinics in remote areas all over the state of California, using telemedicine. Based on a selected sample of key informant interviews with local staff physicians, this study provides further insight into the current process of e-referral which uses electronic communication for making referrals to specialty care. It also identifies key services for telemedicine in primary and specialty care settings within the San Francisco public health system. This study concludes with proposals for a framework that seek to increase collaboration between the referring primary care physician and specialist, to prioritize institution of these key services for telemedicine.

## 1. Research Question

*Primary questions:*

1. to determine key services for telemedicine in primary care and specialty care settings of the San Francisco public health system;
2. to determine a framework within the safety net to prioritize institution of key services for telemedicine.

## 2. Background and Significance

Safety-net settings across the country have struggled with providing adequate access to specialty care services. To explain, a 2002 case study conducted in five cities in the United States found that capacity was strained for specialty care services with some cities reporting wait times of up to a year for types of specialty care services [1]. While a country-wide problem in general particularly in California where patients report having difficulties in obtaining appointments for specialty care services. A 2002-2003 telephone survey of medical directors of all 101 Federally Qualified Health Centers in the state found that 85% of respondents reported that their uninsured patients “often” or “almost always” had difficulties in obtaining specialty care; 41% reported similar difficulties for Medicaid patients [2].

San Francisco has had to deal with the same issue. As the primary safety-net hospital for the City of San Francisco, SFGH is committed to providing specialty care for all the city's residents regardless of their ability to pay, including the city's uninsured and the Medicaid and Medicare patients. Although it was not long until the advent of e-referral, San Francisco General Hospital's electronic specialty care referral system was characterized by a process called “triage by hassle,” [3] which refers to the extra effort both primary and specialty care providers had to make in order to expedite the specialty care referral process of a patient with an urgent condition. The extra effort of the providers in this case would often take the form of numerous handwritten referrals, faxes, phone calls, and pages. Thus, variation in the length of wait times, coupled with poor communication between primary care and specialty care providers in this setting, presents a challenge to the delivery of specialty care services to uninsured and Medicaid patients using the San Francisco public health system [4].

The challenge of access to specialty care services at the primary safety-net hospital, SFGH, is slated for an even greater strain, with a potential increase in the influx of the volume of patients in the San Francisco safety net. In 2006, the City and County of San Francisco instituted a new ordinance which created Healthy San Francisco (HSF), the city's healthcare program for uninsured resident [5, 6]. Since 2007, enrolment in HSF, based on estimates, has grown to over 43,000 uninsured San Francisco adult residents; this equates to a total of 72% of the population in the city [7]. Even though the city has expanded the provider network to include other hospitals and a national HMO (Kaiser Permanente), the strain on the system is slated to get worse with about 26% of all enrollees in the program reporting a delay in medical care or treatment since joining the program [8].

Thus, there currently exists an opportunity to unravel the effects of the HSF expansion on the healthcare safety-net. With this opportunity comes the understanding that the effect on access to specialty care services is an area worthy of further investigation.

Furthermore, in 2006 the California policy environment took a new turn with the passage of Proposition 1D. This educational facilities bond now provides funds for capital improvements to expand and enhance medical education programs in the state [9]. In essence, with this proposition and additional funds, SFGH will be able to connect UCSF and SFGH with clinics in remote areas all over the state of California using telemedicine. The goal of the San Francisco General Hospital and Trauma Center Telemedicine Network is to develop a sustainable and robust urban telemedicine network that facilitates clinical programs to increase access for primary and specialty care patients with a wide range of chronic illnesses in diverse communities throughout the Bay Area, Northern and Central California [10].

### 2.1. Study Aims

**AIM 1**

To determine key services for telemedicine in primary care and specialty care settings of the San Francisco public health system.

This proposition will provide resources for building the basic infrastructure to increase capacity for expanding broadband connections between the hospital and the nexus of outpatient clinics with healthcare providers, both specialists and primary care providers all over the city.

However, it is still unclear as to how SFGH Hospital leadership should use these resources within the context of providing valuable services to clinics in the safety net, alongside enhancing the medical education program using telemedicine Thus, it is clear that the opportunity exists to determine priorities for the use of infrastructural funds to enhance telemedicine services for uninsured and Medicaid patients in the San Francisco public health system.

**AIM 2**

To determine a framework within the safety net to prioritize institution of key services for telemedicine.

## 3. Hypothesis

This study sought to understand and identify key services for telemedicine in primary care and specialty care settings of the San Francisco public health system. Based on a selected sample of key informant interviews with local clinical physician staff, we also sought to determine a framework within the safety net to prioritize institution of key services for the use of infrastructural funds to enhance telemedicine services in primary and specialty care areas of the San Francisco public health system.

## 4. Study Design

Key informant ethnographic qualitative interviews were conducted with a selected convenience sample of stakeholders in targeted roles input from whom was identified as being important to the implementation of key services for telemedicine in the safety net.

### 4.1. Ethnographic Research Design

The study design involves the qualitative method which employed the collection of ethnographic data' during in-depth semi-structured interviews with the selected convenience sample. The qualitative ethnographic approach was employed in this study to examine the nature of the technology-based interaction [11], that is, “black box” interaction with the use of telemedicine, and to identify the sociocultural challenges and barriers with its use in the safety net. 

### 4.2. Ethnographic Method Adopted

The fundamental ethnographic method adopted for this study involved key informant ethnographic qualitative interviews.

### 4.3. Ethnographic Sampling

Participants for the key informant ethnographic interviews were identified and selected as a convenience sample by the Chief Medical Officer of the San Francisco General Hospital and Trauma Center. The program coordinator of the study communicated with the selected individuals to make arrangements to set up the interview in the clinic setting. In order to have been selected as a participant in this study, the following criteria had to be met, Medical Director of the affiliated COPC clinics, and/or the Clinical Chiefs of the UCSF medical specialty divisions. Participation required the willingness to provide written informed consent to be interviewed at the clinic of their choosing to discuss key services for telemedicine in the delivery of specialty care to uninsured and Medicaid patients in the San Francisco public health system. Copy of the recruitment script is attached in Appendix B.

### 4.4. Collection of Ethnographic Data

Study participants for the interviews were identified by the Chief Medical Officer of San Francisco General Hospital and Trauma Center. Once identified, the program coordinator of the study communicated (primarily by electronic mail and secondarily by telephone call) with the selected individuals to make arrangements to provide informed consent forms and set up the interview in the clinic setting. Key informant interviews with Medical Directors of the sampled clinic sites occurred at each specific clinical site under study (see Appendix A). 

Once scheduled, the study coordinator then travelled to each study site and conducted the key informant interviews with the Medical Directors of the clinic sites. The format of the interview has been described in the key informant interview guide (see Appendix C).

The intended format of the interview comprised firstly an initial general introduction focused on an assessment of clinical and administrative operations of the clinic (“tell me about your clinic”), with a keen intention to move towards a case-based scenario to focus on clinic-specific issues. The goal was to introduce a general case-based scenario and to direct provider thoughts regarding specific clinical and operational issues encountered when accessing specialty care services in the safety net. The intention was to then direct providers thoughts to discuss the potential use of telemedicine and to allow for an open-ended discuss ion on the key services for telemedicine that would benefit the provider in their practice.

The overall intent of the interview was to gauge clinic site-specific services for telemedicine and examples of how the use of infrastructural funds for telemedicine would benefit the site.

The semi-structured interviews were designed to take no more than 30 minutes. A sound recording of the conversation was made; however, no name or identifying information was used. 

### 4.5. Ethnographic Analysis

Interviews were recorded with the explicit consent of participants. Recordings were then transcribed verbatim. Transcripts were examined with regard to domains of interest to include but not to be limited to relevant emergent sociocultural constructs. As is standard in qualitative research, an iterative process of analysis was used to examine and re-examine the facts and meanings contained in the data to develop successively more refined ideas.

Clinical and administrative operations of the clinic were assessed, with a keen intention to move towards a case-based scenario to focus on clinic-specific issues as it relates to accessing specialty care services within the safety net. The goal was to introduce a general case-based scenario and to direct provider thoughts regarding specific clinical and operational issues encountered when accessing specialty care services in the safety net. The intention was to then direct providers thoughts to discuss the potential use of telemedicine and to allow for an open-ended discussion on the key services for telemedicine that would benefit the provider in their practice.

The goal was to assess the priorities and identify key services for telemedicine that would benefit from the use of infrastructural funds to enhance specialty care for uninsured and Medicaid patients in the San Francisco public health system.

Specifically, this was assessed by a process of analysis which involved firstly the parsing of descriptive data (text from observation and interview transcripts) according to themes as outlined above and secondly, the development of a set of taxonomic principles (a coding manual for significant sociocultural concepts) and subsequent classification (coding) of those themes and finally, the identification of associations between themes.

Firstly, the text data was read and formulated into 10 to 15 categories that encompass various domains of interest. Once the categories were standardized, all text data was coded. Once all of the textual data had been coded, text segments that referred to particular domains were retrieved. The extracted text segments were then reread and developed into more fine-grained analytical categories to understand what underlied the concerns. Once contextual factors were identified, central themes were investigated further to see what links, if any, exist between themes.

As a result of the ethnographic analyses of the provider interviews, salient themes revealed a set of services for telemedicine which would benefit from the use of infrastructural funds within the safety net.

### 4.6. Validity and Reliability in Ethnographic Research

Problems of reliability and validity have been explored thoroughly by experimenters and other qualitative researchers in the literature. Reliability in ethnographic research is dependent on the resolution of both external and internal design problems [12]. While external reliability depends on whether researchers discover constructs in similar settings, internal reliability refers to the degree to which others would match them with data in the same way as did the original researcher. While reliability is concerned with the replication of scientific findings, validity is concerned with the accuracy. Establishing validity requires determining the extent to which conclusions effectively represent empirical reality and assessing whether constructs devised by researchers represent or measure the categories of human experience that occur [12, 13]. While the internal validity refers to the extent to which scientific observations and measurements are authentic representations of some reality, external validity addresses the degree to which such representations may be compared legitimately across groups. Although reliability and validity are common problems shared by all ethnographers and researchers, results of ethnographic research can often be regarded as unreliable and lacking validity and generalisability. The purpose of this study is not to question or assess the very method of inquiry but to enlist the defined methods of inquiry in understanding the use of telemedicine to develop strategies in the context of the San Francisco Safety-net.

## 5. Ethical Considerations

### 5.1. Informed Consent Process

Participation entailed giving written permission to collect data in the interview settings. Participants in the key informant interviews were provided with a written consent form (see Appendix B) and were then reminded of the consent process at the time of the interview (see Appendix C). 

### 5.2. Subject Confidentiality

Key informant interviews with providers were voice-recorded but stripped of any names or other identifying information in the transcriptions; however, even though participants were informed of the risk that their responses could be shared with the Hospital leadership team at SFGH for the ultimate goal of improving care and services offered, this was not required. Although the tapes and transcriptions have been kept in a locked cabinet on premises at San Francisco General Hospital, Building 20, Suite 2300, and San Francisco, Calif, once this research article is published, they will be destroyed.

### 5.3. Risks and Benefits Analysis

There were few, if any, risks to participants in study. Participation could have resulted in a loss of privacy. The interviews were voice-recorded for the purposes of creating a computer transcription for a word processing program. Care was taken to eliminate names from the transcripts; however, responses from the key informant interviews could have been shared with the leadership team at San Francisco General Hospital with the ultimate goal of improving care and services offered; however was not required. The sound recording was stored in a locked file. Each person in this study had their own code number so that no one other than me and the co-investigators of the study knew who was in my notes. The key to the code of names was kept in a separate locked file and is to be destroyed following completion and publication of the study. The survey data was stripped of any personal identifiers. The potential benefits to the participants were the opportunity to participate in research and contribute to the scientific community by providing greater understanding in low resource healthcare settings and assist in further enhancements and developments of the telemedicine program for the San Francisco safety net. 

### 5.4. Payments to Participants

Participants in the key informant interviews did not receive any payments, nor any form of remuneration for their time. They were informed of this during the informed consent process as detailed in the C HR (Committee on Human Research) application.

### 5.5. Cost to Subjects

There were no costs to participants in this study other than their time for participating in the key informant interviews.

## 6. Study Population

The study population for the key informant interviews were twenty participants to include the Medical Directors of 14 Community-Oriented Primary Care Clinics (COPC) and the 6 UCSF medical specialty division clinics at San Francisco General Hospital and Trauma Center (SFGH). The study population for this study, although small in number, was sufficient to assure the validity and reliability of this ethnographic research since they were identified as key stakeholders for telemedicine, input from whom was defined as being vitally important to the implementation of key services for telemedicine in the safety net.

The list of these study sites is shown in Appendix A. The study population included those that were invited to participate in the study.

### 6.1. Participant Inclusion Criteria

To be included in the study, a study participant had to have been a Medical Director/Clinical Chief affiliated with COPC clinics and/or the UCSF medical specialty divisions at SFGH. Participants must have also voluntarily given written informed consent to discuss telemedicine in their practice for the delivery of specialty care to patients.

### 6.2. Participant Exclusion Criteria

Participants were ineligible to join the study if they had not voluntarily given written informed consent to participate in the interview. Informed consent was obtained at two points during the study, once during the recruitment process (Appendix B) either through email or phone or again at the time of the interview.

## 7. Measurement Tools

The interview guide for the key informant interviews was created with assistance from the Global Health Sciences research methods class materials and qualitative/ethnography analysis experts at UCSF, Global Health Sciences Division at 50 Beale Street. The goal of the interview guide was to elicit as much information as possible regarding clinical and administrative operations of the clinic site in general, with a keen intention to move towards a case-based scenario to focus on clinic-specific issues as it relates to accessing specialty care services within the safety net and to then direct providers thoughts to discuss the potential use of telemedicine and to allow for an open-ended discussion on the key services for telemedicine that would benefit the provider in their practice. A copy of the interview guide has been included in Appendix C.

## 8. Results and Discussion

A total of 11 qualitative interviews were conducted with primary and specialty care providers from eleven different clinics throughout the safety net. Out of the 11 providers, 8 were Medical Directors of the COPC and 3 were Chiefs of UCSF Medical Specialty clinics. Repeated attempts to contact the remaining 6 PCP and 3 Specialists were accomplished in order to include them in the study; however none were included. Even though some of the providers noted interest in participation and arranged for a time at their convenience for the interview, last minute schedule changes as well as cancellations resulted in nonparticipation.

The purposes of these interviews were to gain an in-depth understanding of the current process of patient referral to specialty care services within the safety net and to improve the delivery of these types of services to patients using telemedicine. Key informant interviews engaged with physicians in a general conversation regarding information on their current practice and experience with the electronic referral process, with a keen focus on ways in which improvements could be made using types of services or programs using telemedicine technology. Semi-structured interviews ranged in duration between 30 and 60 minutes each and were conducted in the specific primary and specialty care clinic settings.

Preliminary qualitative data reveals several key themes pertinent to the study. 

### 8.1. Current Process of Electronic Referrals (E-Referral)

The current system of making specialty care referrals between the PCP and the specialists were based on an electronic platform embedded into the EMR. A majority of the providers noted their satisfaction and enthusiasm with the e-referral system and that the institution of this novel approach to accessing specialty care resulted in decreasing the amount of time it took primary care patients to obtain a first time appointment with a specialist at SFGH.

Some quotes from providers to illustrate this point:

*“E-referral has allowed us as primary care providers to have better communication with specialists.”*

*“It has helped us (Specialists) decrease our patients' wait times for an Appointment in our clinic.”*

*“Great thing about e-referral in my opinion (PCP) is that it is a conversation starter. In the past I would refer patients to rheumatology and you would have to wait till the rheumatologist saw you before you could have a conversation about it … and with e-referral you are told that you may need to get a CT scan before you see the specialist.”*

*“The e-referral system has significantly improved our process; tremendous amount of improvement resulting in a significant improvement in our wait times, from nine months to about two months.”*

*“I found e-referral to be quite efficient for me, especially as you build a library ofresponses.”*

*“E-referral was a fantastic move with this innovation in bringing this type of system here, was because we had a notoriously lost somehow somewhere in the system people's consult request forms, with 40% of patients coming to the clinic and did not have a consultation with them, and we had no idea why these patients were in the clinic, and it was not uncommon for me to page a PCP and ask as to what I should be doing with these patients., and patients would tell me different stories and the PCP's would have a different story and e-referral closed that loop.”*

Even though e-referral has worked tremendously well in vastly improving access to specialists and reduced wait times for an appointment, interviews with providers also revealed some gaps in the system.

Some quotes from providers to illustrate:

*“E-referral is just a mechanism.”*

*“Understanding the e-referral and its intentions, it has worked well to make the referral process much easier and doing some consultation and pre-visit consultation, and almost perhaps has avoided unnecessary visits, that has seemed to work well … But actually getting them (specialists) to tell us (PCP) what has happened, and making the feedback loop in the system and making the system more user friendly for the patient, that has remain unchanged.”*

*“We should all have one central medical record, and the specialist and the PCP should have access to all the same information and we should not have paper and that there is no reason in today's age for me (PCP) to write a note that nobody else can see.”*

*“It would be great to have just like the e-referral system gives you feedback on the front end on e-referral where you end up finding out right away, we will see your patient or not, or order your tests and then get back to me, its once the patient actually sees the specialist and after the patient sees the specialist, that is when the system begins to break down.”*

*“Right now, we have no way of knowing if a patient was actually seen (in the specialty clinic); until I see the patient the next time, and ask the patient if they could make it to their appointment.”*

*“Sometimes, we can tell if a patient was registered and did not actually complete a visit (specialty), but only if they place a note in the LCR or fax a note to us, but very rarely occurs.”*

*“Different clinics (specialty) are better at putting a note in the LCR, and some are not. It's very patch work and not uniform, different clinics have different ways of handling that.”*

*“The biggest challenge with the e-referral system is being able to get data or records from some of the outpatient primary care clinics which may not necessarily have the same system as we do, so we are not able to receive that information from our LCR system. It has been a challenge to ensure that our patients come in with that type of information, whether they are in our system or not. We are working very hard to ensure that the media by which we communicate with the primary, has the necessary information that we need transfer to the patient and that the patient brining in that information with them.”*

*“The patient shows up to see me, they do not actually know what's going on with them, and I look at the LCR and do not see a note and so then I have to spend considerable amount of time either calling the (specialty) clinic and asking someone to fax me the note … which is not as simple as … “Hey I need this” they*'*re like wait a minute, let me go look, and ask me to hold on, and that … or I have to send an interdepartmental request for information to medical records department to fax over the notes … so I do not have any real time access and cannot do any real time work and I do not have the data I need to it to follow up or to help the patient out.” *

*“E-referral has incredible capacity to make changes, but it would need a whole lot of more infusion of support to see its potential, otherwise it has the risk of becoming primarily a tracked referral system with appointment time setting, and less of what the original vision, which would be a modality for training and education of providers, improving care … it does improve care on many levels, but I do not think it reaches its full capacity because if the clinics are getting e-referral reviewers faced with 25–50 e-referral requests per day.”*

Overall, comments from the interviews revealed gaps in the referral process. Examples of these gaps include (1) inconsistent feedback loops back to the PCP after conducting a specific specialty visit, (2) Receiving medical records from a variety of specialty clinics in different electronic or paper formats, and (3) the inability to efficiently track if a patient does not show up for their subspecialty appointments unless otherwise noted in the patient visit history section of the LCR.

**Patient “No Shows”**

Majority of primary care clinics track their patient “No show” rates; however this is done in a nonstandardized manner. As a step in the direction of keeping track of the “no shows” as well as maintaining control over the process improvement, patients not showing up for their appointments with specialists were being tracked by an MEA or Registered Nurse or in certain circumstances, the Medical Director of the clinic track this information and record it into an excel spreadsheet or a sheet of paper. The San Francisco City-Wide, COPC Quality Improvement Committee decided to focus on reducing their “no show” rate as part of their annual performance improvement initiative and to work towards optimizing effective use of their resources. A major concern with this change in the process was that medical evaluation assistant and ancillary staff faced constant barriers in trying to obtain access to the e-referral system due to restrictions in its use amongst the nonclinical community.

In addition to these central themes in the gaps, other challenges included the need to order certain diagnostic workups in advance of the specialist visit. Even though diagnostic tests such as specialized procedures (e.g., GI Colonoscopy/Endoscopy) for high need, high impact specialties are pushed to be completed in advance of the specialty visit, additional review is usually required for these types of e-referrals. This additional review proved to delay a patient from being able to effectively complete the specialist visit process which in turn caused a delay in follow-up care at the primary care level. At times, these additional checks also proved to be a deterrent for patients to complete the initial visit to the hospital, affecting their ability to complete a specialist referral.

Quote to illustrate a concern with a provider:

*E-referral has not allowed us to have appointments any earlier than before, and in the case of certain specialties, GI more particularly, it has made it difficult for us to have patients be seen, Gastroenterology as a specialty is much less likely to schedule an appointment for a specialty referral and is probably because they have been overwhelmed and have become much more selective and I am sure it is evidence based. The criteria for patients getting a colonoscopy are much more selective than other specialties, and within our system, no one can get a screening colonoscopy, we just do not have the resources for that. In this case, a screening colonoscopy would be important for the early detection of colorectal cancer, for which one of the standards of care is to obtain a colonoscopy once every ten years, but we do not have the resources to do that, so we use a faecal occult blood test every year. We have patients who have a family history or have had a previous colonoscopy where polyps were found and for those patients it is where we err on the conservative side, and ration care.*

Moreover, a PCP may also not be aware if a patient may or may not have completed these pre- and postvisits for the diagnostic workup or specialists visit until six to eight months later in certain circumstances.

In addition, patients may also be scheduled for these types of visits too soon at times, resulting in appointment letters being mailed out too soon and possibly resulting in patients not showing up for the specialist visit.

A quote to illustrate:

*We are committed to checking e-referrals for our clinic (specialty) 3 days a week at a very minimum and I think it happens on a daily basis and we can book people into our clinic as quickly as the following week. So, in fact when we first launched e-referral the first 6–9 months, our no show rates increased for a period of time. I do not have formal numbers for this, but we really noticed a creep up of our no shows, but then we found out that we were reviewing e-referrals so quickly, booking their appointments so quickly, and patients never got any letter for their appointment until after their appointment dates passed. So, now we are more routinely booking people two weeks out even though we could take them in faster.*

### 8.2. Key Recommendations for Improvements in E-Referral

 Key recommendation for an improvement in the current system includes the expansion of e-referral to increase collaboration between PCP and the Specialist. This can be accomplished by the following:

1. facilitating the initiation of referrals from the PCP to the specialists;
2. incentivising the specialists to provide more timely feedback to the PCP during the e-referral process itself and to include feedback after the onsite subspecialty visit;
3. reducing barriers for both the PCP and specialist to centrally track patients who do not show up for their appointments (no Shows), or for some reason or the other decide to leave the clinic before being seen by the specialist (come and go's).

Key informant interviews with providers revealed a brief list of examples of e-referral improvements. A summary of the specific examples of the ways in which an enhanced level of collaboration between the PCP and the specialist could be accomplished is shown in Table 1.

Some of the interviews also revealed that some subspecialty groups at SFGH were working on a provider-referral algorithm which would likely introduce more reliability within the current system and ultimately define set turnaround time frames and approved guidelines for certain type of specialty referrals. There is a task force or a work group that is currently reviewing this algorithm as a potential improvement to the e-referral system.

### 8.3. Videoconferencing Technology for Telemedicine

When PCP and specialists were asked about the use of videoconferencing technology during the referral process, a variety of mixed opinions were expressed.

Some quotes to illustrate:

*“Making e-referral more interactive with videoconference may not be as valuable as you think it may, since it will inevitably impact my patient flow and is not worth it. The interesting thing about e-referral is that you do at the end of the visit … Say for example you are with the patient for 15 minutes and in the last 2-3 minutes you place an e-referral … You have already budgeted time to be with the patient … And if then at that time you decide or know of the option to videoconference in to increase the interaction in e-referral, then it will have already impacted the patient flow.”*

*“We have many Spanish speaking patients, so, we could use videoconferencing technology for language interpreters and that would be important.”*

*“Physical examinations would be hard to do through videoconferencing technology, but they could get the process started, build a connection with the patient so that they could feel more comfortable to see the specialist at some point.”*

*“A majority of the patients referred to our specialty would require the need for the patient to complete a thorough physical evaluation with any of us, before a patient could be referred to a diagnostic test or even a follow up visit. The same exists with patients requiring other specialist services.”*

*“Videoconferencing works beautifully for translation … You could consider doing it instead of clinic spots instead of having them come to you … but I'm not sure if that would be doing the patient a service, this could be a different service we could provide, but it would be a separate clinic and would be called telemedicine clinic.”*

However, videoconferencing technology may be useful for those patients who may not ordinarily show up for a routinely scheduled appointment or for those patients living in “hard to reach” areas of the city.

For example, a primary care provider summarizes,

*“In situations where patients who are psychiatrically impaired or have mobility issues and come in for evaluation of a rare GI Mass or a tumour or complex rheumatologic case wherein a group of symptoms may need to be reviewed and a patient may not be in a position to make it to his or her appointment, video conferencing technology may vastly improve their access to a specialist.”*

Although mixed opinions were voiced regarding the possibility of using videoconferencing technology as part of the e-referral process, this type of technology is already in use in the safety net via the language interpretation service (VMI) which is currently being expanded to different areas of the public healthcare system. Based on existing data, 40 percent of the patients seen in our system would require some sort of language interpreter service

In the words of one of the providers,

*“We could start with VMI technology as a starter since we have a group of patients who would benefit tremendously from having another person interpret language and the potential to build on its existing infrastructure to provide access to more services would be beneficial.”*

The safety net is already becoming familiar with this type of technology, which means an easier transition in utilizing this type of technology for telemedicine. In addition, majority of the revenue for this purpose could be invested in the videoconferencing technology itself, and building technical capacity for the purpose of education and training with utilisation of the new technology could be made earlier under the auspices of VMI scale up within the safety net.

“We have made it clear that we could provide consultation that is not patient Specific, as in general question…”

### 8.4. Types of Telemedicine Services

The interviews revealed that providers, both primary care and specialists, favored the use of education-based activities as a key service for the use of telemedicine technology. Such education-based activities would span the following domains: *Specialist to PCP, Specialist to patient, *and *Specialist to PCP and Patient *(real time). (A summary of the types of telemedicine services can be found in Table 2.)

#### 8.4.1. Specialist to Primary Care Provider Telemedicine

One of the key rationales for education-based activities as a key service for the use of telemedicine technology was not having enough specialists to span the entire system to meet demand, and as such the idea of sharing the knowledge base over a vast catchment area would prove to be the most beneficial use of telemedicine technology. One idea discussed was having the usual “*Grand Rounds*” broadcasted throughout the safety-net clinics. Since grand rounds encompass the wide spectrum of general scenario-based disease-specific case studies, broadcasting these sessions would prove to expand the knowledge base across the safety net without reinventing the wheel.

Quotes to illustrate:

*“Grand rounds getting funnelled into a conference room (Carr Auditorium), sitting in front of a TV in our clinic and having providers remotely watch could be very helpful.”*

*“I think it*'*s a great idea to be able to have our providers and staff at the clinic assembles in the conference room to be able to watch grand rounds during lunch time.” *

*“Webcast lectures would be great, anyone being able to log in and connect to Carr Auditorium”*

*“We (PCP) could be hooked up to a conference room and cases could be brought to them (specialists).”*

*“Educating the providers on all fronts, PCP and specialists as to we offer and what services and what specialty services are necessary and to really assist them in making these types of decisions.”*

*“Assisting the PCP in managing their patients and potentially avoiding the referrals where they could have been addressed should they have had the information, simplifying the referral process so that PCP and their eligible patients have easier access to care, and the process of the referral is simpler.”*

Some of the PCPs supported this idea, and in fact a few primary care providers were ready to enlist a specific area of the clinic for such to occur as a “noon time lunch activity.” Although, not all of the providers supported this idea, they were optimistic that such access to specialist provider and education would prove beneficial in the long run.

#### 8.4.2. Specialist to Patient Telemedicine

Primary care providers felt that patients in the primary care setting would benefit from a specialist education session in the group format. The group session would not only serve as a forum for patients with similar disease groups to interact with the specialist clinician, but would also serve as a supportive group atmosphere for patients to identify with each other.

Quoting a provider with this idea:

*There are some patient education programs that could be incorporated, we currently have an education program for Hepatitis B and C patients, there are some types of liver diseases we could provide patient education programs ahead of time assisting patients in making decisions about pursuing an appointment with the specialist primarily for the treatments purposes versus being followed by the PCP.*

Due to the financial premium of having a specialist conduct these sessions themselves and the time commitment involved, UCSF, SFGH, and DPH could identify interested qualified personnel within its already extensive resource of APN healthcare professionals and redefine the role of the Advanced Practice Nurse, the Nurse Practitioner, Physician Assistant, Certified Nurse Leader, and the Certified Nurse Specialists. However such an approach based on task shifting would have to be contingent on the patient's voice and the political buy-in and leadership support of the CNO as well as would require the necessary approvals from the organizational structures put in place by the Nursing Executive Committee (NEC) and the Committee on Interdisciplinary Practice (CIDP).

Quoting providers with this idea,

*“There is a lot of potential here (with telemedicine) and the other question is that do you need doctor to do it … we have huge number of nurses who have advance training here in San Francisco general hospital, nurse leaders, nurse mangers, nurse specialists there are people who have advance educational training so should there be a physicians doing the education or should they be one of these advance practice nurses who does that … that depends upon what the patient wants and what the primary provides wants.”*

This type of telemedicine service based on education would focus on preparing the patient for the physical and mental rigors of presenting at the specialist appointment, in addition to self-care education focusing on lifestyle modification, dietary and nutritional education, and education on managing chronic pain.

Quoting another provider with this idea,

*“I have heard of a taskforce convened and they have started talking about Group visits for GI Patients and preparation for a colonoscopy could help and would also help increase access to care and assist in preparation in advance of the visit … a place to obtain the informed consent, with risk and benefits to be explained as well as more information from the specialist … This would be the initial visit … and all of this could be done in a group setting by either a specialist, but more importantly since it is education, Nurse practitioner, Physician Assistant or maybe even a Registered Nurse.”*

Key informant interviews with providers also revealed interest in educational activities focused on (chronic) disease management for HIV, diabetes, and asthma.

Some quotes to illustrate this:

*“The core of our patient population are people who are or were homeless at some point, HIV inflicted, lots of mental illness and active substance use, multiple chronic conditions, chronic pain being one of those and people who in general have had very variable experiences with the health system in general.”*

*“We have a more African American diabetic population, average number of hypertensive's, higher number of HIV positive patients ranking third amongst ten, and take care a lot of kids … we are a very busy clinic within the system.”*

Educational sessions to prepare patients for the specialist visit in areas of highest need included group liver sessions (hepatitis B, hepatitis C, viral hepatitis) and Group cardiology sessions (anticoagulation education, cardiology medication education, cardiac risk factor education, cardiac valve disease education)

One providers view:

*“What would be neat is if we could do group visit stuff with our patients, for example if we got 20 patients and if I connected remotely with some specialist services, so patients do not have to end up going to the general, they could just come here, and meet with me since they are already comfortable coming to the clinic here, Hepatitis C classes I believe is something they have talked about doing.”*

#### 8.4.3. Specialist to PCP and Patient Telemedicine

One of the key rationales for this type of clinical/diagnostic-based activity as a key service for the use of telemedicine technology was having patients who would otherwise not have adequate access to specialty care services at the hospital, due to their inability to physically travel to the hospital or generally lack in following up on PCP recommendations due to discomfort or unfamiliarity with the specialty care setting or for patients undergoing transitional issues between primary and acute care or specialty settings.

Some quotes from providers below:

*Patients have a lot of questions and are emotionally distressed about the risk of a certain procedures, and as a PCP I do not have access to that type of information, which the patient would like to know” In many ways in trying to facilitate what should happen at the specialty clinic … And this patient will come to me, and he is like, he will go to SFGH and never make a decision … I cannot make a decision until I see you (PCP) again, they*'*re going to do this operation and I want to know what you think. Who am I (as PCP) to make this decision, I am not the specialist who is going to cut into your spinal cord…” How do we cut that step out … Patient feeling like the PCP needs to be in on the situation … This is an example of a reason for me as the PCP being in the same room” “Sometimes specialist may write that off as an uninterested patient … But as a PCP, I feel the duty to translate that for the specialist, and if I can help translate that, we can move the process forward. *

*I would find it tremendously helpful if we could use telemedicine to do rounds or when there is one of my patients in the hospital, and they are very complicated patients with social and discharge disposition issues that come up, what I end up having to do, is to page the resident, the attending or the social worker, and stay near the phone and returning calls at various times … if I think it's important enough, I will come into the hospital and have a meeting with the patient, the in house team and myself and perhaps the case manager and myself to work out the discharge plan. It would smooth transitions from the hospital to the community for particularly challenging patients; the in patient team could consult with the outpatient team to work on issues of transitions.*

Although dependant on a case by case basis, certain “hands free” specialties not requiring touch or cases which would allow for a consultation to occur without mandatory physical presence may render telemedicine technology useful.

A quote from a provider:

*“Certain sub specialties lend themselves better to real time telemedicine, I would imagine dermatology or psychiatry would be a great example of telemedicine in real time, rashes, skin conditions, conversations, you can take photos of everything, and in this case, photos could be very helpful in this circumstance, but maybe not the most perfect example.”*

## 9. Study Limitations

This study does not come without limitations. Firstly, the study population is very small and as such, the small sample size utilized in this study calls into question the controversial nature of the validity and reliability traditionally contested in ethnographic research. As a result, care must be taken when interpreting the findings of this study to ensure the sample size is taken into consideration. Secondly, although it was initially envisioned to have follow up surveys to be sent to the interviewees after conducting the key informant interviews, it was later decided to not administer the survey since there was very little interest in responding to a survey and fear of a very low response rate. Thirdly, engaging the physicians and clinical staff during the interview sessions proved to be quite challenging since it was hard to conceive what could be accomplished with telemedicine without providing concrete examples to guide the interview. Future studies should ensure such examples are prepared in advance of the interview to assist in engaging and guiding the discussion effectively. Finally, a major limitation of the study was that the interview defined a telemedicine scenario for the providers explicitly towards the end of the interview as “videoconferencing,” but then asked participants to consider ways in which telemedicine might be a useful tool in practice and with referrals, thus biasing the concept of telemedicine as videoconferencing and planting this notion in their minds, resulting in suggestions as those mentioned such a dermatology and psychiatry. A follow-up study useful in this context would be to go back to the group and show them a demonstration of the various ways in which telemedicine has been used—the full breadth of its capacity—and then register ideas for telemedicine implementation, to bolster current findings.

## 10. Next Steps and Future Work

This qualitative study employing ethnographic methods used a selected sample of primary and specialty care providers to gain an in-depth understanding of the current process of patient referral to specialty care services within the safety-net and to improve the delivery of these types of services to patients using telemedicine. The rationale for the inclusion of these types of methods in the study was to ensure the involvement of the actual stakeholders in the decision making process. Conducting qualitative interviews with the PCP and specialists also served as a means of obtaining general consensus and community buy-in, potentially resulting in the eventual high rate of acceptance of the strategy developed as well as influence on the behaviour change. The results of the study provide insights and key implications on work and patient flow patterns within the primary and specialty care clinics of the safety net.

Key recommendations focus on technical enhancements to the current e-referral interface as well integrating key telemedicine services into the operational and institutional infrastructure of the safety net. Based on the framework proposed, it is recommended that the institution of these changes be adopted within the existing operational and institutional infrastructure of the safety net with little or no additional operational revenue other than the funds required for technology inputs into the system as well as prioritize the institution of these key services for telemedicine by sharing the results with the appropriate work groups and committees within the SFDPH and UCSF network for appropriate followup.

Future work in this area should focus on the understanding of these types of services for telemedicine as supplementary to the provision of direct medical care, the role of the Advanced Practice Nurse in the provision of educational, and initial point of care specialty counselling for patients requiring a higher level of care and the inclusion of the perception of the patient's voice as well as the assessment of the providers acceptance of telemedicine technology. In addition, the longitudinal cost effective analyses of using the telemedicine approach as a modality would eventually assist in the creation of a business plan for opportunities to scale up and spread these services to other safety-net settings similar to the San Francisco context.

## Figures and Tables

**Table 1:** Increased collaboration between the PCP and specialist.

Referral from PCP	Feedback from specialist	Centralized tracking for “no shows” and “come and go's”/reminders
Ex: video file option (MPEG) with speech recognition transcription software embedded into electronic referral system. Requires recording apparatus such as a webcam on the computer. *This could occur in presence of patient or PCP alone. *	Ex: expanding library of responses with the option of customizing free text linked to the medical literature/pub med databases for automated citations of peer-reviewed literature*. Possibility of saving time and improving efficiency and reliability in design. *	Ex: automated phone call reminders “one day” in advance of visit, “no show” and “come and go” tracking within e-referral for both PCP and specialist.

**Table 2:** Summary of the three types of telemedicine services.

Specialist to PCP	Specialist to Patient (*Specialist can also take the place of APN: RN, CNL, CNS, NP, PA) *	Specialist to PCP & Patient (Post patient “hand off” by PCP)
*Education/General Scenario/Case based*	*Education/General Scenario based in Group format*	*Clinical/Diagnostic/Real*-*time Consultation *
Grand Rounds	Chronic Disease Management (HIV/Diabetes)	Disease specific—based on case by case basis “Patient Rounds”
Provider education (CME)	Group Visits with patients in preparation for specific diagnostic procedure (Ex. Informed consent for colonoscopy, Cardio tests, other procedures)	“Hands-free” specialties “Does not require touch” Ex: Psychiatry and Dermatology

**Table 3:** Study sites/population of interest.

COPC Sites (14)	UCSF Medical Specialty Division Clinics at SFGH (9)
(1) Castro-Mission Health Center	(1) Breast
(2) Chinatown Health Center	(2) Cardiology
(3) Curry Senior Center	(3) Endocrinology
(4) Housing and Urban Health Clinic	(4) Gastroenterology
(5) Maxine Hall Health Center	(5) Hepatology
(6) Ocean Park Health Center	(6) Liver
(7) Potrero Hill Health Center	
(8) Silver Avenue Family Health Center	
(9) Southeast Health Center	
(10) STD Clinic on 7th Street station (PEP)	
(11) Special Programs for Youth	
(12) Tom Waddell Health Center	
(13) Transgender Clinic	
(14) Women's Health Center	

## Appendices

### A. Study Sites/Population of Interest

For more details see Table 3.

### B. Written Informed Consent

Study Title Increasing Access to Specialty Care Services in the San Francisco Healthcare Safety-net: Determining the Needs and Priorities for Telemedicine Services from the Provider Perspective

My name is *Ken Russell Coelho *and I am a graduate student researcher in the Global Health Sciences Division at the *University of California, San Francisco*. I am currently working on my graduate fieldwork and Masters of Science thesis under the direction of Principal Investigator, *Dr. Hal Yee, MD, Ph.D*. and faculty advisor, *Dr Wayne Steward, MPH, PhD*, and would like to invite you to take part in my research study, which looks determining the needs and priorities for telemedicine services from the provider perspective with the goal of improving the delivery and access of specialty care to uninsured and Medicaid patients using the San Francisco public health.

Research studies include only people who choose to take part. Please take your time to make your decision about participating, and discuss your decision with others if you wish. If you have any questions, you may ask the researchers of the study.

You are being asked to take part in this study since you have been identified as a key informant Medical Director/Clinical Chief at the affiliated COPC clinics, and/or the UCSF medical specialty divisions. The purpose of the study is to improve access and quality of specialty care services in San Francisco. About 30 clinical providers will take part in this study from a wide variety of primary and specialty care services in the San Francisco Healthcare Safety-net.

If you agree to take part in this research, you will be interviewed for 30 minutes at the location of your clinic/hospital office at a time of your convenience. The session will include your participation wherein you will be asked about your experience in the delivery of specialty care to uninsured and Medicaid patients in your practice. A sound recording of your conversation will be made. We will not use your name or identifying information when we make the copy. However, responses from this interview may be shared with the leadership team at San Francisco General Hospital with the ultimate goal of improving care and services offered. Care will be taken to eliminate your name from the transcripts; although worthy comments which require prompt follow up will be identified to the SFGH leadership team.

After the interview, a computer transcription of the interview will be created. The sound recording will then be stored in a locked file. Each person in this study will have their own code number so that no one other than myself and the co-investigators of the study will know who you are in my notes. The key to the code of names will be kept in a separate locked file and will be destroyed following completion of this study.

Unless prior arrangements are made, the entire study will be conducted at the location of your clinic/hospital office and at a time of your convenience

After the interview, you will be informed of an opportunity to participate in a *brief survey *which will be emailed to you in 4–6 weeks by the Principal Investigator of the study. The survey will take about 5 minutes to complete. The purpose of the survey is to identify key telemedicine services that would benefit from the use of infrastructural funds to enhance specialty care for uninsured and Medicaid patients in the San Francisco public health system.

There are no known risks to you from taking part in this research, and no foreseeable direct benefit to you either. However, it is hoped that the research will benefit the scientific community by providing greater understanding of the use of this new approach in low resource healthcare settings and assist in further enhancements and development.

If the need arises to obtain specific health outcome information, a separate health information consent form will be provided for the informed consent process to occur. The cons ent process complies with the Health Information Privacy & Accountability Act (HIPAA), which protects the privacy of individually identifiable health information, and the confidentiality provisions of the Patient Safety Rule, which protect identifiable information being used to analyze patient safety events and improve patient safety.

Your participation in this research is strictly voluntary. You are free to refuse to take part in the study at any time. You may refuse to answer any questions and may stop taking part in the study at any time. Whether or not you choose to take part in this research will have no bearing on your status at UCSF and your affiliated institution.

If you have any questions about the research, you may contact me Ken Russell Coelho, directly at (415) 206-8539 or by e-mail: ken.coelho@ucsf.edu and/or my faculty advisor Dr. Hal Yee, at (415) 206-4808 or by email: hyee@medsfgh.ucsf.edu.

If you agree to take part in the research, please sign the form below. Please keep the other copy of this agreement for your future reference.

If you have any question regarding your treatment or rights as a participant in this research project, please contact the University of California, San Francisco, Office of the Committee on Human Research at (415) 476-1814, or by email: chr@ucsf.edu.

I have read this consent form and I agree to take part in this research

**Table:**

	
Date	Participant's Signature for Consent
……	………………………………
	
Date	Person Obtaining Consent
……	………………………

### C. Interview Guide for Key Informant Interviews

Thank you for joining me today and agreeing to participate in this Key Informant Interview.

My name is Ken Russell Coelho and I am currently working with Dr. Hal Yee, Chief Medical Officer of San Francisco General Hospital and Director of the Center for Specialty Care Access and Quality at the University of California, San Francisco. I am a graduate student researcher in Global Health Sciences at UCSF and this research study is being conducted as part of my Masters of Science graduate thesis.

We are interested in learning more about your experience as part of the San Francisco Safety-net.

As you are aware, safety-net settings across the country have grappled with providing adequate access to specialty care services, and San Francisco has also had to grapple with the same issue. With the advent of the Healthy San Francisco, this challenge of access to specialty care services at San Francisco General Hospital has presented even greater strain, with an anticipated potential increase in the influx of the volume of patients. Interestingly, the California Policy environment took a new turn with the passing of Proposition 1D. This educational facilities bond will provide funds for capital improvements to expand and enhance medical education programs in the state, with a focus on telemedicine. In essence, with this proposition and additional funds, SFGH will be able to connect UCSF and SFGH with outpatient clinics using telemedicine.

As a result, we are particularly interested in your ideas on how to improve the delivery of specialty care to uninsured and Medicaid patients in the San Francisco public health system, with the use of telemedicine.

We hope that during this conversation you will be able provide us with information on your current practice and to learn from you about your experience in your practice and ways we can improve the types of services and programs offered.

The length of this interview will be 30 minutes and we will tape record the session and then we will use the tape to make a written copy of the comments. We will not use your name or identifying information when we make the copy. However, responses from this interview may be shared with the leadership team at San Francisco General Hospital with the ultimate goal of improving care and services offered. Care will be taken to eliminate your name from the transcripts; although worthy comments which require prompt follow up will be identified to the SFGH leadership team.

You may refuse to answer any questions and may stop taking part in the study at any time. Whether or not you choose to answer any question or participate, this will have no bearing on your status at UCSF and your affiliated institution.

Do you have any questions for me? (Address any questions that may be asked or refer them to PI if needed.)

Begin recording.

Questions/Points of conversations.

Let's start with: Introduction (General Questions about their clinic, Niceties).

Now, I would like to start with a general scenario which you may or may not find typical presenting at your clinic (*INSERT CASE SCENARIO—varied based on PCP or specialist OF PATIENT IN PRIMARY CARE/SPECIALTY CARE SETTING PRESENTING WITH SYMPTOMS NECESSITATING NEED FOR SPECIALTY CARE SERVICE REFERRALS/CARE AT SFGH*).

1. Does this scenario sound familiar to your Clinic/practice? Please feel free to elaborate.
2. From your experience, what is the current process for this patient? Could you walk me through some of the steps encountered?
3. What do you like about the current process?
4. What do you NOT like about the current process?

*As specific steps in the process are identified, probe as to which steps are considered for the best and which have been for the worse.*
(5) What in your opinion could improve this process (in general)? Please elaborate.

I am now beginning to get a sense of the unique issues faced in your practice.

1. Now, what if you had the opportunity to change ONE thing in your practice to improve this process of making referrals/providing consult services, do you have any thoughts on what those change would be?

Now, I would like you to go back to the initial scenario which you may or may not find typical presenting at your clinic (*INSERT CASE SCENARIO—varied based on PCP or specialist OF PATIENT IN PRIMARY CARE/SPECIALTY CARE SETTING PRESENTING WITH SYMPTOMS NECESSITATING NEED FOR SPECIALTY CARE SERVICE REFERRALS/CARE AT SFGH*).

Now, imagine, it is 10am and your patients “present with these same symptoms necessitating a referral to specialty care service, BUT now you are now able to VIDEOCONFERENCE in and consult with the PCP/Specialists/Fellow all at the same time.

1. How would this change the process in your clinic/site? Please elaborate.
2. Can you walk me through some of the changes?
3. Do you like this change?
4. What do you NOT like about this change?

*As specific steps in the process are identified, probe as to which steps are considered for the best and which will worsen with this new approach.*
(5) Can you think of any barriers to implementing such a telemedicine program at your clinic site?
(6) What are your thoughts on:
  - Maintenance of the equipment for the program? Please elaborate.
  - Maintaining a medical record? Please elaborate.
  - Reimbursement? Please elaborate.

Specific to primary care providers:

1. If you had the opportunity of collaborating with and were provided access to any type of *telemedicine services*, if available, with the specialists at SFGH, do you have any thoughts on what some of those services would look like?

Specific to the specialists:

1. If you had the opportunity of collaborating with or were provided access to any type of *telemedicine services*, if available, with the Primary Care Provider's in the outpatient setting, do you have any thoughts on what some of those services would look like?

Thank you very much for your time and an engaging discussion. This has been an interesting learning experience for me. We hope to use this information to ultimately make improvements in the delivery of specialty care to uninsured and Medicaid patients, and your contribution to this pursuit is greatly appreciated.

You will be receiving an email from my PI, Dr Hal Yee in the next couple weeks or so. Please do respond to the survey by rating your selections on types of telemedicine services you would find most useful in your practice.

Once again, if you have any questions about this research study or any questions asked, feel free to contact me Ken Russell Coelho, directly at (415) 206-8539 or by e-mail: ken.coelho@ucsf.edu and/or my faculty advisor/Principal Investigator, Dr. Hal Yee, at (415) 206-4808 or by email: hyee@medsfgh.ucsf.edu.

Have a great day!

Interview guide adapted from the Telecommunications and Information Policy Institute document, Needs and Assets Assessment tool, University of Texas-Austin (http://www.utexas.edu/research/tipi/research/needs_assest_assestments.pdf) Accessed on March 9th 2010.

## Abbreviations

APN: Advanced Practice Nurse
CHR: Committee on Human Research
CIDP: Committee on Interdisciplinary Practice
COPC: Community Oriented Primary Care
CNO: Chief Nursing Officer
CNS: Certified Nurs e Specialist
CNL: Certified Nurse Leader
EMR: Electronic Medical Record
FHC: Family Health Center
GMC: General Medical Clinic
GI: Gastroenterology
HMO: Health Maintenance Organization
HSF: Healthy San Francisco
LCR: Lifetime Clinical Record
MEA: Medical Evaluations Assistant
NEC: Nursing Executive Committee
PCP: Primary Care Provider
SFGH: San Francisco General Hospital
SFDPH: San Francisco Department of Public Health
UCSF: University of California, San Francisco
VMI: Videoconferencing medical interpretation.
